# Prevention of LPS-Induced Acute Kidney Injury in Mice by Bavachin and Its Potential Mechanisms

**DOI:** 10.3390/antiox11112096

**Published:** 2022-10-24

**Authors:** Ka-Yun Ban, Ga-Young Nam, Donghee Kim, Yoon Sin Oh, Hee-Sook Jun

**Affiliations:** 1 College of Pharmacy and Gachon Institute of Pharmaceutical Sciences, Gachon University, Incheon 21936, Korea; 2 Lee Gil Ya Cancer and Diabetes Institute, Gachon University, Incheon 21999, Korea; 3 Department of Food and Nutrition, Eulji University, Seongnam 13135, Korea; 4 Gachon Medical Research Institute, Gil Hospital, Incheon 21565, Korea

**Keywords:** acute kidney injury, lipopolysaccharide, bavachin, oxidative stress, inflammatory response

## Abstract

Acute kidney injury (AKI) is a serious complication of sepsis with a rapid onset and high mortality rate. Bavachin, an active component of *Psoralea corylifolia* L., reportedly has antioxidant, anti-apoptotic, and anti-inflammatory effects; however, its beneficial effects on AKI remain undetermined. We investigated the protective effect of bavachin on lipopolysaccharide (LPS)-induced AKI in mice and elucidated the underlying mechanism in human renal tubular epithelial HK-2 cells. Increased serum creatinine and blood urea nitrogen levels were observed in LPS-injected mice; however, bavachin pretreatment significantly inhibited this increase. Bavachin improved the kidney injury score and decreased the expression level of tubular injury markers, such as neutrophil gelatinase-associated lipocalin (NGAL) and kidney injury molecule-1 (KIM-1), in both LPS-injected mice and LPS-treated HK-2 cells. LPS-induced oxidative stress via phosphorylated protein kinase C (PKC) β and upregulation of the NADPH oxidase (NOX) 4 pathway was also significantly decreased by treatment with bavachin. Moreover, bavachin treatment inhibited the phosphorylation of MAPKs (P38, ERK, and JNK) and nuclear factor (NF)-κB, as well as the increase in inflammatory cytokine levels in LPS-injected mice. Krüppel-like factor 5 (KLF5) expression was upregulated in the LPS-treated HK-2 cells and kidneys of LPS-injected mice. However, RNAi-mediated silencing of KLF5 inhibited the phosphorylation of NF-kB, consequently reversing LPS-induced KIM-1 and NGAL expression in HK-2 cells. Therefore, bavachin may ameliorate LPS-induced AKI by inhibiting oxidative stress and inflammation via the downregulation of the PKCβ/MAPK/KLF5 axis.

## 1. Introduction

Sepsis is a systemic inflammatory response caused by a viral, fungal, or bacterial infection [1]. Sepsis progression causes multiple organ dysfunction and can ultimately lead to death [2]. Excessive release of inflammatory cytokines in sepsis causes extensive tissue damage and fatal organ dysfunction [3]; therefore, prompt and appropriate management is required for the prevention and treatment of sepsis.

Acute kidney injury (AKI) is a major complication of sepsis and is characterized by a sharp decline in renal function [4]. The development of AKI during sepsis increases patient morbidity and mortality rates. These are associated with an increased length of stay in the intensive care unit, hence consuming considerable healthcare resources [5]. However, there is limited information on sepsis-induced AKI and the appropriate therapeutic methods are still highly debatable. A decrease in glomerular filtration rate and an increase in serum creatinine and blood urea nitrogen (BUN) levels are characteristic features of AKI progression [6]. Additionally, the expression of novel biomarkers of AKI, such as neutrophil gelatinase-associated lipocalin (NGAL) and kidney injury molecule-1 (KIM-1), increase in the urine and plasma [7]. Acute tubular necrosis and dysfunction of tubular epithelial cells are also involved in the development of sepsis-induced AKI [8].

Lipopolysaccharide (LPS) is an endotoxin present on the surface of gram-negative bacteria, and infusion/injection of LPS has been widely used as a model of experimental sepsis-associated AKI [9]. LPS is released into the blood circulation during sepsis and binds to toll-like receptor (TLR)-4 expressed in renal tubular epithelial cells, which can contribute to AKI pathogenesis, escalating oxidative stress, inflammatory responses, tubular cell death, renal hypoperfusion, low blood pressure, and consequently a gradual decrease in kidney function [10,11].

Plant-derived medicines have attracted considerable attention owing to their minimal side effects and cost-effectiveness. Many natural compounds, such as flavonoids, polyphenols, and terpenoids, are effective against AKI because of their anti-oxidative and anti-inflammatory effects [12]. However, it is not yet clear whether these natural compounds can be used as drugs or dietary supplements to prevent or treat AKI. Bavachin, an active component of *Psoralea corylifolia* L., has been reported to have antioxidant and anti-inflammatory effects [13]. Recently, it was reported that bavachin attenuates LPS-induced inflammatory response and inhibits the activation of the NRLP3 inflammasome in macrophages [14]. However, the effect of bavachin on sepsis-associated AKI has not been investigated. Therefore, we investigated the effects of bavachin on LPS-induced AKI and its underlying molecular mechanisms.

## 2. Materials and Methods

### 2.1. Animal Model

Eight-week-old male C57BL/6 mice were purchased from Orientbio (Seongnam, Korea) and housed at standard temperature (22 ± 2 °C) and humidity (50 ± 5%) under a 12 h/12 h light/dark cycle. We determined the treatment doses of 10 and 20 mg/kg bavachin based on previous studies investigating the antioxidant effects in mice [15]. The mice were randomly assigned to four groups: control (control; *n* = 10), vehicle + LPS (vehicle + LPS; *n* = 10), bavachin 10 mg/kg + LPS (bava 10 + LPS; *n* = 10), and bavachin 20 mg/kg + LPS (bava 20 + LPS; *n* = 10). Bavachin (Chengdu Must Bio-Technology Co., Ltd., Chengdu, China) or vehicle was orally administered via gastric cannulation once daily for seven days. Subsequently, the sepsis-associated AKI was induced by an intraperitoneal injection of LPS (10 mg/kg in 0.9% saline; LPS from *Escherichia coli* O111:B4; Sigma-Aldrich, St. Louis, MO, USA), which is widely used [16,17,18,19]. An equal volume of 0.9% saline was intraperitoneally injected into the control group. Mouse urine was obtained from metabolic cages 24 h before and after LPS injection. All mice were sacrificed 24 h after LPS injection to obtain the kidney and blood samples. After removing the renal capsule, the right kidney was immediately frozen at −80 °C and the left kidney was immediately fixed in 10% neutral buffered formalin. Serum was separated from the blood samples by centrifugation and evaluated for serum creatinine and BUN levels. All animal experiments were approved by the Institutional Animal Care and Use Committee of the Lee Gil Ya Cancer and Diabetes Institute, Gachon University (LCDI-2020-0121).

### 2.2. Renal Histological Assessment and Immunostaining

Kidney tissues were fixed, embedded in paraffin, and sectioned into 4 μm sections. To analyze pathological changes, kidney sections were stained with hematoxylin (#1.05175.0500; Sigma-Aldrich) and eosin (MA0101015; BBC, McKinney, TX, USA) (H & E). To analyze proximal tubular injury, kidney sections were stained with periodic acid-Schiff (PAS) (K1433-30; Biovision, Milpitas, CA, USA). Tubular injury was scored based on the percentage of injured area with a slight modification of the method in a previous report [16]. Injury was scored as follows: 0, no damage; 1, injured area 1–10%; 2, injured area 11–25%; 3, injured area 26–75%; and 4, injured area 75% or more.

For immunohistochemical staining (IHC), kidney sections were subjected to heat-induced antigen retrieval using citrate buffer (CR2072-100-60; Biosesang, Seongnam, Korea) (10 mM citric acid, 0.05% Tween 20, pH 6.0) at 100 °C for 40 min and permeabilized using 0.2% Triton X-100 in PBS for 10 min. The sections were treated with Protein Block Serum-Free Ready-To-Use solution (Dako North America, Inc., Carpinteria, CA, USA) at 25 °C for 1 h and incubated with primary antibodies at 4 °C for overnight. The following primary antibodies were used: NGAL (ab63929; Abcam, Cambridge, UK), KIM-1 (NBP1-76701; Novus Biologicals, Centennial, CO, USA), NADPH oxidase 4 (NOX4, ab109225; Abcam), malondialdehyde (MDA, ab243066; Abcam), Krueppel-like factor 5 (KLF5, ab137676; Abcam), 4-hydroxynonenal (4-HNE, MAB3249; R & D Systems, Minneapolis, MN, USA), protein kinase C (PKC) β (#46809; Cell Signaling Technology, Inc., Danvers, MA, USA), and phospho-PKC (p-PKC) β (#9371; Cell Signaling Technology, Inc.). Immunohistochemical staining was performed using a Polink-2 Plus horseradish peroxidase (HRP) Rabbit with a DAB kit (D39-18; GBI Labs. Inc., Bothell, DC, USA) to detect NGAL, KIM-1, NOX4, PKCβ, and p-PKCβ, or the Polink-2 Plus Mouse HRP using a DAB Kit (D37-18; GBI Labs. Inc.) to detect 4-HNE and MDA according to the manufacturer’s protocol. Nuclei were counterstained with hematoxylin (Sigma-Aldrich). The sections were subsequently mounted and observed under an AXIO Imager Z1 microscope (Carl Zeiss Inc., Oberkochen, Germany) at the Core-facility for Cell to In-vivo imaging. IHC quantification of 4-HNE and MDA was performed using ImageJ software (version 1.51; National Institutes of Health, Bethesda, MD, USA) [20].

### 2.3. Biochemical Analyses in Blood

Serum levels of creatinine and urea nitrogen were analyzed commissioned to the SEMI (Busan, Korea). Serum cytokine levels were measured using a Quantikine ELISA kit for tumor necrosis factor-α (TNF-α) (MTA00B), interleukin-6 (IL-6) (M6000B), and interleukin-1β (IL-1β) (MLB00C) (R & D Systems). All assays were performed according to the manufacturer’s instructions.

### 2.4. Cell Culture

The human renal tubular epithelial cell line HK-2 was obtained from the American Type Culture Collection (ATCC, Rockville, MD, USA). HK-2 cells were maintained in RPMI-1640 (LM001-05; Welgene, Daegu, Korea) containing 5% fetal bovine serum (16000-044; Gibco™, Waltham, MA, USA) and 1% penicillin/streptomycin (FS202-02; Welgene) at 37 °C in an atmosphere of 5% CO_2_ in 95% air. HK-2 cells were seeded at a density of 2.5 × 10^5^ in 60 mm dishes and incubated for 18 h. In the preliminary experiment, to determine the dose of bavachin, 0.1 μg/mL was the most effective concentration to inhibit LPS-induced activation of NF-κB. Therefore, we chose 0.1 μg/mL bavachin for in vitro experiment. Cells were pretreated with vehicle or 0.1 μg/mL bavachin for 1 h, followed by 1 μg/mL LPS for 1, 1.5, or 15 h. Equal amounts of dimethyl sulfoxide (DMSO) for bavachin and distilled water for LPS were used as vehicles.

### 2.5. Reactive Oxygen Species (ROS) Measurement

HK-2 cells (5 × 10^3^/well) were seeded in 96-well black plates (#6005020; PerkinElmer Inc., Waltham, MA, USA), incubated for 2 d, pretreated with 0.1 μg/mL bavachin for 1 h, and then treated with 5 μg/mL LPS for 1.5 h. Cells were washed with PBS and incubated with 10 μM DCFH-DA (C6827; Thermo, Waltham, MA, USA) for 15 min. Intracellular ROS levels were measured using a VICTOR Nivo™ multimode plate reader (PerkinElmer Inc.).

### 2.6. Quantitative Real-Time Polymerase Chain Reaction (qRT-PCR)

Total RNA was lysed from kidney tissues or cells using the RNAiso Plus reagent (#9109; Takara Bio Inc., Kusatsu, Japan), and the concentration and purity were calculated. cDNA was prepared using a PrimeScript First Strand cDNA Synthesis Kit (6110A; Takara Bio Inc.), and qRT-PCR was performed on a CFX384^TM^ Real-Time PCR System (Bio-Rad, Hercules, CA, USA) using SYBR Premix Ex Taq II and ROX plus (Takara Bio Inc., Shiga, Japan). Cyclophilin or 18s ribosomal RNA (18s RNA) were used as the reference genes, and the relative amounts of mRNA were calculated using the 2^−ΔΔCT^ method. The gene-specific primer pair sequences used in the qRT-PCR are listed in Table 1.

### 2.7. Western Blotting

Proteins were extracted and Western blotting was performed, as previously described [17]. Equal amounts of lysate samples (10–30 µg total) were separated by SDS-PAGE for probing with the following primary antibodies: NGAL (ab125075), KLF5, MDA, NOX4, KIM-1, Abcam; PKCβ, P-PKCβ, nuclear factor-kappaB (NF-κB) P65 (#4764), P-NF-κB P65 (Ser536) (#3033), P-p38 MAPK(Thr180/Tyr182) (#4511), P38 MAPK (#9212), P44/42 MAPK (ERK1/2) (#9102), P-P44/42 MAPK (ERK1/2) (Thr202/Tyr204) (#9101), SAPK/JNK (#9252), and P-SAPK/JNK (Thr183/Tyr185) (#9251), Cell Signaling Technology, Inc.; β-actin (sc47778; Santa Cruz Biotechnology, Inc., Santa Cruz, CA, USA). Then, the membranes were incubated with HRP-conjugated secondary goat anti-rabbit (Jackson ImmunoResearch, West Grove, PA, USA) or goat anti-mouse (Invitrogen, Waltham, MA, USA) antibodies for 1 h at 25 °C. The target protein bands were visualized using Immobilon-Western chemiluminescent HRP substrate (Millipore Corp., Billerica, MA, USA) and the LAS4000 imaging system (Fujifilm Corp., Tokyo, Japan) or Amersham ImageQuant 800 system (Cytiva Life Sciences (formerly GE Healthcare Life Sciences), MA, USA). Luminescence was quantified using ImageJ software.

### 2.8. Transient Transfection

For small interfering RNA (siRNA) transfection, HK-2 cells (2.5 × 10^5^ cells) were plated in 60 mm dishes and transiently transfected with 10 pmol KLF5 siRNA (Bioneer Inc., Daejeon, Korea) or scrambled siRNA (Bioneer Inc.) using Lipofectamine RNAiMAX (#13778150; Invitrogen) according to the manufacturer’s instructions. After 6 h, cells were treated with 1 μg/mL LPS for 1.5 or 15 h.

### 2.9. Statistical Analysis

The significance of differences among groups was analyzed by one-way analysis of variance (ANOVA) with Tukey’s multiple comparison test using GraphPad Prism version 7.03 (GraphPad Software Inc., San Diego, CA, USA). All results are presented as the mean ± standard error of the mean (SEM). Statistical significance was set at *p* < 0.05.

## 3. Results

### 3.1. Bavachin Restores Histological Changes and Kidney Function in LPS-Induced AKI Mice

**Figure 1:**
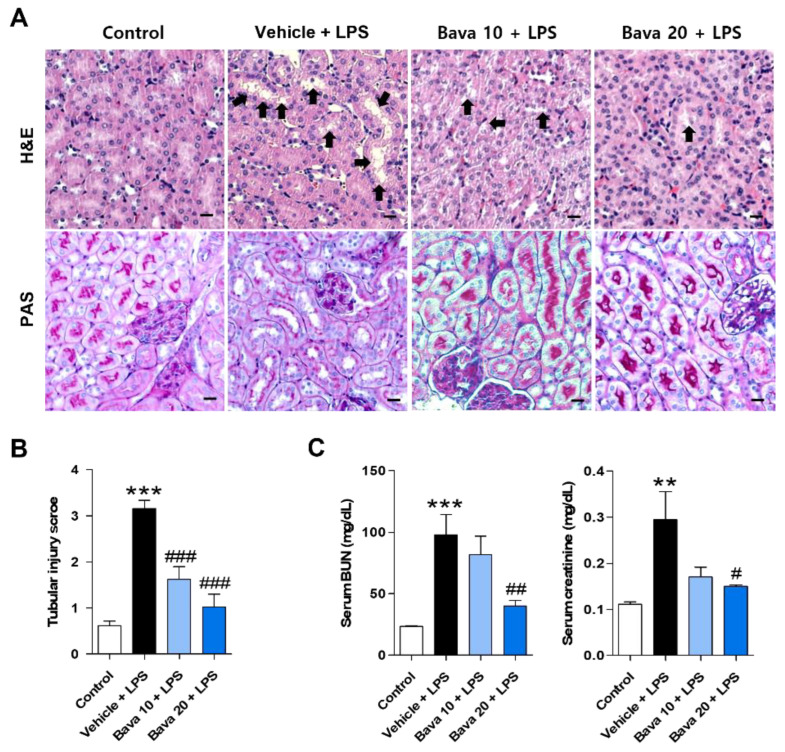
Bavachin restores histological changes and kidney function in LPS-induced AKI mice. After 24 h of LPS injection, the mice were sacrificed and kidney tissue and blood were collected. (**A**) Representative images of hematoxylin and eosin (H & E) and periodic acid-Schiff (PAS) staining of the kidney tissues. Nuclei were counterstained with hematoxylin (original magnification, 200×; scale bars, 20 μm; *n* = 3 mice per group). The black arrow in the H & E-stained section indicates degeneration and vacuolization of renal tubular epithelial cells. (**B**) Tubular injury was scored based on the percentage of injured area as follows: 0, no damage; 1, injured area 1–10%; 2, injured area 11–25%; 3, injured area 26–75%; and 4, injured area 75% or more. (**C**) Serum levels of blood urea nitrogen (BUN) and creatinine were analyzed (*n* = 6 mice per group). Data are presented as mean ± standard error of the mean (SEM). ** *p* < 0.01, *** *p* < 0.005 vs. control; ^#^ *p* < 0.05, ^##^ *p* < 0.01, ^###^ *p* < 0.005 vs. vehicle + LPS.

To evaluate the histological changes in LPS-induced AKI mice by bavachin treatment, kidney tissues from each group were stained with H & E and PAS. Histological abnormalities, such as renal tubular vacuolation and tubular cell necrosis, were observed in LPS-injected mice, and the administration of bavachin attenuated LPS-induced structural injury (Figure 1A). Next, the glycocalyx of the proximal tubule brush border [18,19] was stained with PAS to investigate the brush border loss. PAS stained cells (strong pink color) were markedly decreased in LPS-injected mice, but were clearly increased by bavachin treatment (Figure 1B). The levels of BUN and serum creatinine, which are biomarkers of renal function, were significantly increased in LPS-injected mice, and these levels were decreased by 10 mg/kg bavachin treatment, although not significantly, and were significantly decreased by administration of 20 mg/kg bavachin (Figure 1C). These results indicate that bavachin ameliorates structural injury and kidney dysfunction in LPS-induced AKI mice.

### 3.2. Bavachin Decreases the Expression of Tubular Injury Markers in LPS-Induced AKI Mice and LPS-Treated HK-2 Cells

**Figure 2:**
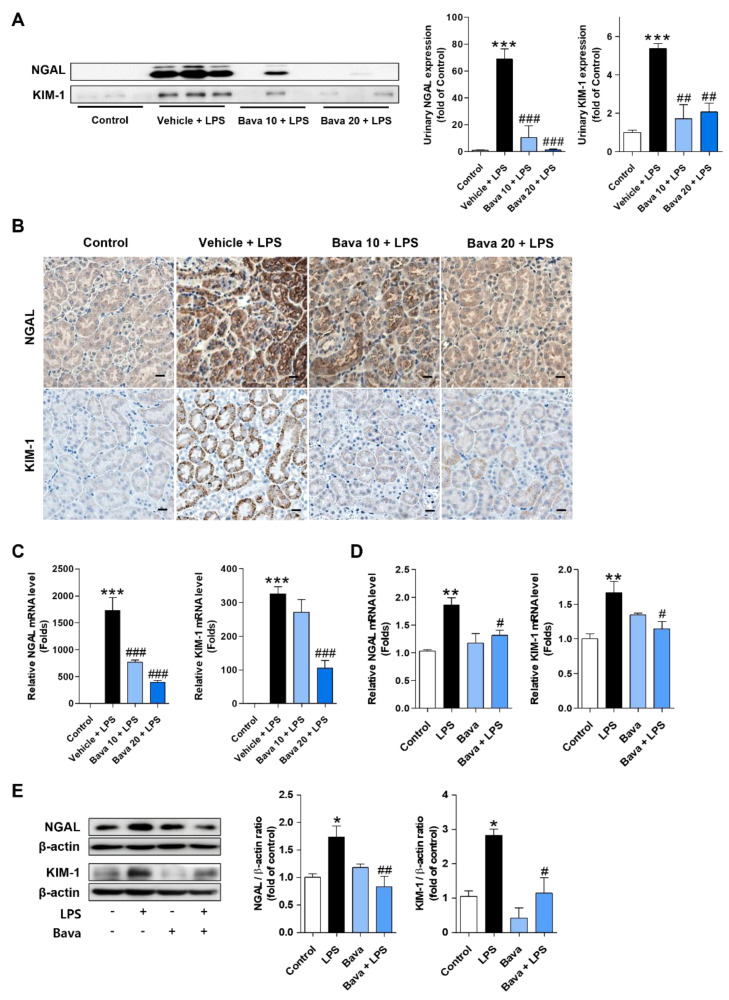
Bavachin decreases the expression of NGAL and KIM-1 in LPS-induced AKI mice and LPS-treated HK-2 cells. (**A**–**C**) After 24 h of LPS injection, mice were sacrificed and kidney tissues and urine were collected. (**A**) The urine protein levels of neutrophil gelatinase-associated lipocalin (NGAL) and kidney injury molecule-1 (KIM-1) were analyzed by Western blotting and quantified using ImageJ software (*n* = 3 mice per group). (**B**) Immunohistochemical detection of NGAL and KIM-1 was performed in kidney tissues using DAB. Nuclei were counterstained with hematoxylin (original magnification, 200×; scale bars, 20 μm; *n* = 3 mice per group). (**C**) The mRNA levels of NGAL and KIM-1 in the kidney tissues were analyzed by qRT-PCR (*n* = 5 mice per group). (**D**,**E**) HK-2 cells were pretreated with 0.1 μg/mL bavachin for 1 h and then treated with 1 μg/mL LPS for 15 h. (**D**) The mRNA levels of NGAL and KIM-1 were analyzed by qRT-PCR (*n* = 3). (**E**) Protein levels of NGAL and KIM-1 were analyzed by Western blotting, quantified using ImageJ software, and normalized to β-actin (*n* = 3). Data are presented as mean ± SEM. * *p* < 0.05, ** *p* < 0.01, *** *p* < 0.005 vs. control; ^#^ *p* < 0.05, ^##^ *p* < 0.01, ^###^ *p* < 0.005 vs. vehicle + LPS or LPS.

As we observed tubular cell injury by LPS and restoration by bavachin pretreatment, we investigated the expression levels of NGAL and KIM-1, widely used as tubular injury markers, in urine and kidney tissues of mice. Urinary protein levels were assessed by Western blotting. The two biomarkers were expressed at very low levels in the control group and a significant increase was observed after LPS injection; however, administration of 10 or 20 mg/kg bavachin significantly reduced these levels (Figure 2A). Immunohistochemistry also showed the same trend as Western blotting; NGAL and KIM-1 were highly expressed in the tubular epithelial cells of kidney tissues from LPS-injected mice and the bavachin treatment significantly reduced the staining density, especially at 20 mg/kg (Figure 2B). Moreover, increased mRNA expression of NGAL and KIM-1 in kidney tissues from LPS-injected mice was observed, but this induction was significantly attenuated by bavachin pretreatment (Figure 2C). As we observed that the increased expression of tubular injury markers in the kidneys of LPS-injected AKI mice was attenuated by bavachin treatment, we wanted to confirm this effect in HK-2 cells (proximal tubular epithelial cells). HK-2 cells were pretreated with bavachin and treated with or without LPS for 15 h and the expression levels of NGAL and KIM-1 were measured. As shown in Figure 2D, the mRNA expression levels of these genes were significantly increased by LPS treatment compared with control cells, but treatment with bavachin significantly suppressed the expression levels (Figure 2D). Consistent with this, bavachin treatment suppressed the increase in NGAL and KIM-1 protein levels in LPS-treated HK-2 cells (Figure 2E). Treatment with bavachin alone did not show any changes in the expression of these genes at either the mRNA or protein level (Figure 2D,E).

### 3.3. Bavachin Decreases ROS Production in LPS-Induced AKI Mice and LPS-Treated HK-2 Cells

**Figure 3:**
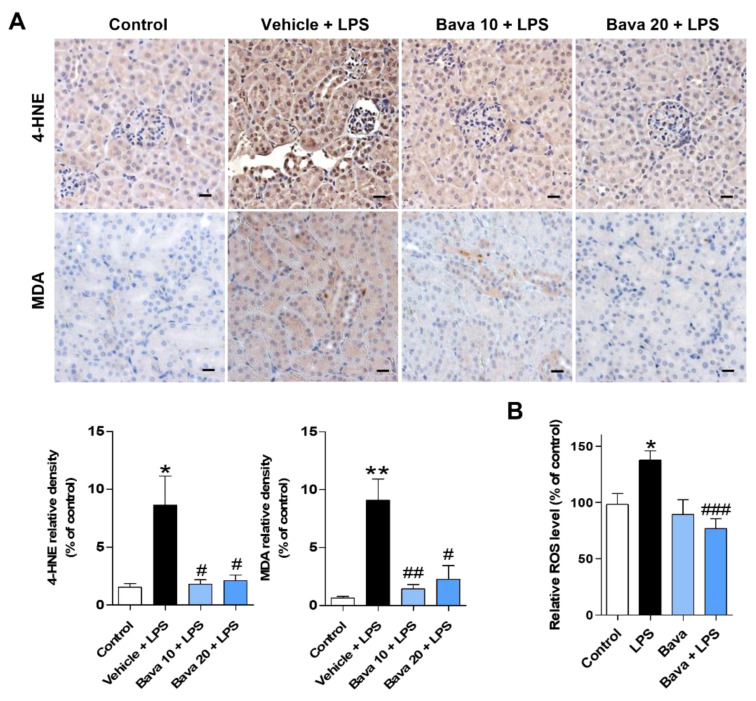
Bavachin decreases ROS production in LPS-induced AKI mice and LPS-treated HK-2 cells. (**A**) Immunohistochemical detection of 4-hydroxynonenal (4-HNE) and malondialdehyde (MDA), as markers of ROS generation, was performed in kidney tissues using DAB. Nuclei were counterstained with hematoxylin (original magnification, 200×; scale bars, 20 μm; *n* = 3 mice per group). Bottom: Relative expression densities of 4-HNE and MDA quantified using ImageJ software. (**B**) HK-2 cells were seeded in a 96-well black plate, pretreated with 0.1 μg/mL bavachin for 1 h, and then treated with 5 μg/mL LPS for 1.5 h. Intracellular ROS levels were measured using a VICTOR Nivo™ multimode plate reader using a fluorometric assay with DCFH-DA. The relative levels of ROS by DCF fluorescence intensity were compared to those in the control. Data are presented as mean ± SEM. * *p* < 0.05, ** *p* < 0.01 vs. control; ^#^ *p* < 0.05, ^##^ *p* < 0.01, ^###^ *p* < 0.005 vs. vehicle + LPS or LPS.

Oxidative stress plays an important role in LPS-induced AKI [21]. Therefore, we investigated the effect of bavachin on LPS-induced renal oxidative stress in AKI mice by immunostaining with antibodies against 4-HNE and MDA, which are well-known byproducts of lipid peroxidation [22,23]. As shown in Figure 3A, 4-HNE and MDA levels were negligible in control mice, but their levels were significantly increased in the kidneys of LPS-injected mice. However, bavachin treatment significantly attenuated LPS-induced ROS generation (Figure 3A). To quantitatively examine ROS levels, we measured ROS levels with or without bavachin treatment using a fluorometric assay with DCFH-DA in LPS-treated HK-2 cells. We found that exposure to LPS alone induced a significant increase in DCF fluorescence, and this increase was prevented by bavachin pretreatment (Figure 3B). To examine whether ROS generation was involved in tubular cell injury induced by LPS, HK-2 cells were pretreated with the ROS scavenger, N-acetyl-cysteine (NAC), and then treated with LPS. We found that NAC pretreatment significantly inhibited LPS-induced NGAL and KIM-1 expression at both the mRNA and protein level (Supplementary Figure S1A,B).

### 3.4. Bavachin Decreases PKCβ Activation and NOX4 Expression in LPS-Induced AKI Mice and LPS-Treated HK-2 Cells

**Figure 4:**
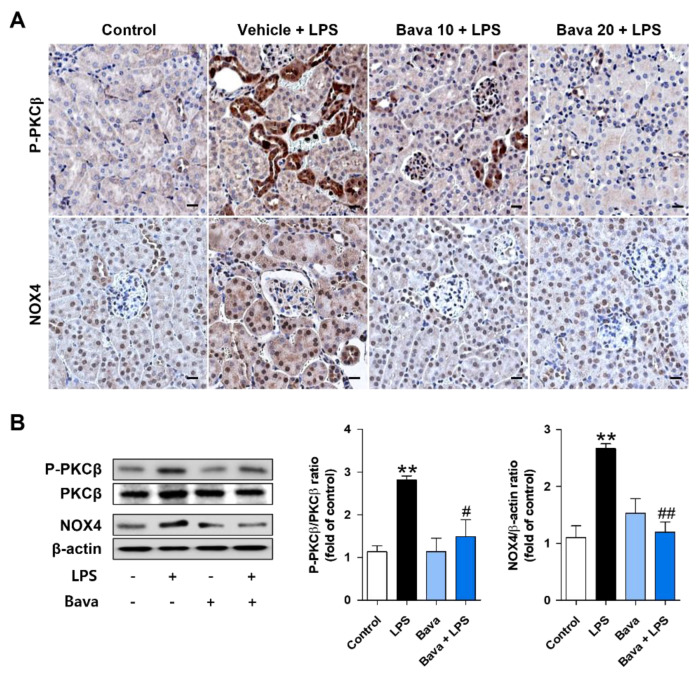
Bavachin decreases PKCβ activation and NOX4 expression in LPS-induced AKI mice and LPS-treated HK-2 cells. (**A**) Immunohistochemical detection of phospho-protein kinase C β (P-PKCβ) and NADPH oxidase 4 (NOX4) in kidney tissues using DAB. Nuclei were counterstained with hematoxylin (original magnification, 200×; scale bars, 20 μm; *n* = 3 mice per group). (**B**) HK-2 cells were pretreated with bavachin 0.1 μg/mL for 1 h and then treated with 1 μg/mL LPS for 1 h. The protein levels of p-PKCβ and NOX4 were analyzed by Western blotting, quantified using ImageJ software, and normalized to PCKβ and β-actin, respectively (*n* = 3). Data are presented as mean ± SEM. ** *p* < 0.01 vs. control; ^#^ *p* < 0.05, ^##^ *p* < 0.01 vs. LPS.

NADPH oxidase (NOX) 4, the major NADPH isoform in the kidney, is involved in redox processes associated with renal disease and is known to be a significant contributor to ROS generation [17,24]. Moreover, PKCβ activation induces NOX4 expression to produce ROS [25,26]; therefore, we examined the expression of the PKCβ/NOX4 axis in the absence or presence of bavachin in LPS-injected mice and LPS-treated HK-2 cells. As shown in Figure 4A, high levels of NOX4 and P-PKCβ proteins were detected in kidney tissues of LPS-injected mice compared with those of control mice; in contrast, bavachin treatment reduced the levels of these proteins (Figure 4A). LPS treatment significantly increased the PKCβ activation and NOX4 expression, which was decreased by bavachin pretreatment (Figure 4B), which is consistent with the findings in the AKI mouse model. These results indicated that bavachin reduced LPS-induced ROS production by inhibiting the PKCβ/NOX4 axis.

### 3.5. Bavachin Decreases the Expression of Inflammatory Cytokines by Downregulating the MAPK/NF-κB Pathway in LPS-Induced AKI Mice and LPS-Treated HK-2 Cells

**Figure 5:**
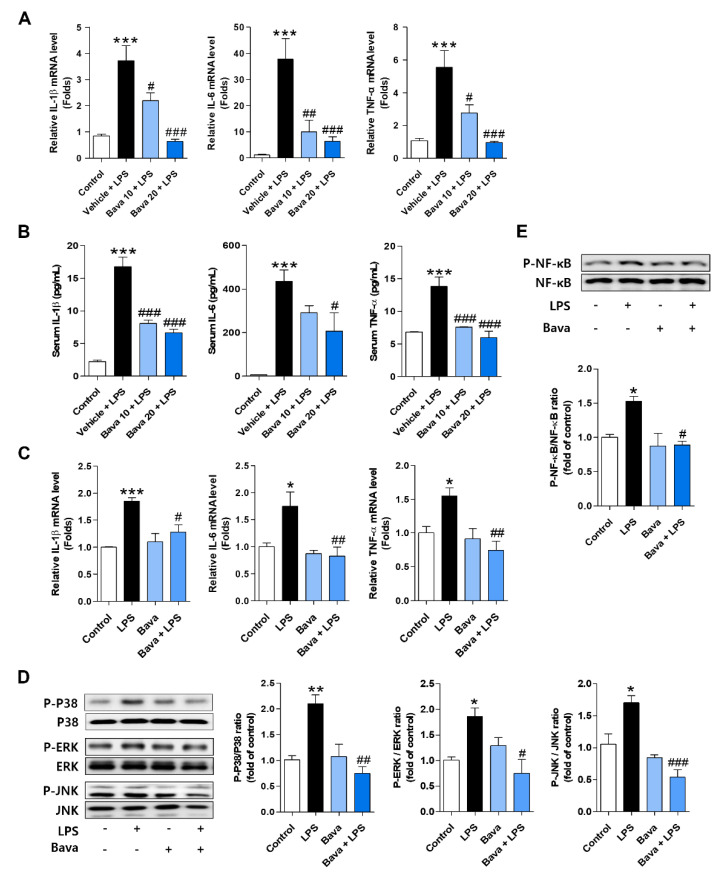
Bavachin decreases the expression of inflammatory cytokines by downregulating the MAPK/NF-κB pathway in LPS-induced AKI mice and LPS-treated HK-2 cells. (**A**) The mRNA levels of interleukin (IL)-1β, IL-6, and tumor necrosis factor-α (TNF-α) in kidney tissues were analyzed by qRT-PCR (*n* = 5 mice per group). (**B**) Protein levels of IL-1β, IL-6, and TNF-α in serum were analyzed by ELISA (*n* = 5 mice per group). (**C**) HK-2 cells were pretreated with 0.1 μg/mL bavachin for 1 h and then treated with LPS for 15 h. The mRNA levels of IL-1β, IL-6, and TNF-α were analyzed using qRT-PCR (*n* = 4). (**D**,**E**) HK-2 cells were pretreated with 0.1 μg/mL bavachin for 1 h and then treated with 1 μg/mL LPS for 1.5 h. (**D**) The protein levels of phospho (P)-P38 (P-P38), P-ERK, P-JNK MAP kinase, and (**E**) phospho-nuclear factor kappa B (P-NF-κB) were analyzed by Western blotting; quantified using ImageJ software; and normalized to P38, ERK, JNK, and NF-κB, respectively (*n* = 3). Data are presented as mean ± SEM. * *p* < 0.05, ** *p* < 0.01, *** *p* < 0.005 vs. control; ^#^ *p* < 0.05, ^##^ *p* < 0.01, ^###^ *p* < 0.005 vs. vehicle + LPS or LPS.

As LPS-induced ROS triggers an acute inflammatory response and bavachin exhibits anti-inflammatory action [14], we investigated these effects using LPS-injected mice and HK-2 cells. As shown in Figure 5A, the mRNA levels of inflammatory cytokines such as IL-1β, IL-6, and TNF-α in kidney tissues were significantly increased by LPS injection, and bavachin administration attenuated the expression of these cytokine mRNAs in a dose-dependent manner (Figure 5A). Consistently, the protein levels of inflammatory cytokines in sera were significantly increased by LPS injection and decreased by bavachin administration (Figure 5B). When we observed this effect in HK-2 cells, intracellular proinflammatory cytokines such as IL-1β, IL-6, and TNF-α were significantly increased 15 h after LPS treatment compared with those in control cells and were significantly reduced by bavachin pretreatment (Figure 5C). The MAPKs and NF-κB pathways are involved in the induction of proinflammatory cytokines [27]. Therefore, we examined the phosphorylation of P38, ERK, JNK, and NF-κB in the absence or presence of bavachin. As shown in Figure 5D, phosphorylated P38, ERK, and JNK were increased in LPS-treated cells and this activation was significantly decreased by bavachin pretreatment (Figure 5D). Finally, the activation of NF-κB was observed in LPS-treated cells and bavachin pretreatment reduced the level of NF-κB activation (Figure 5E). These results indicate that bavachin reduces LPS-induced inflammatory responses through inhibition of the MAPK/NF-κB pathway.

### 3.6. Bavachin Decreases NF-κB Signaling via Down-Regulation of KLF5 in LPS-Induced AKI Mice and LPS-Treated HK-2 Cells

**Figure 6:**
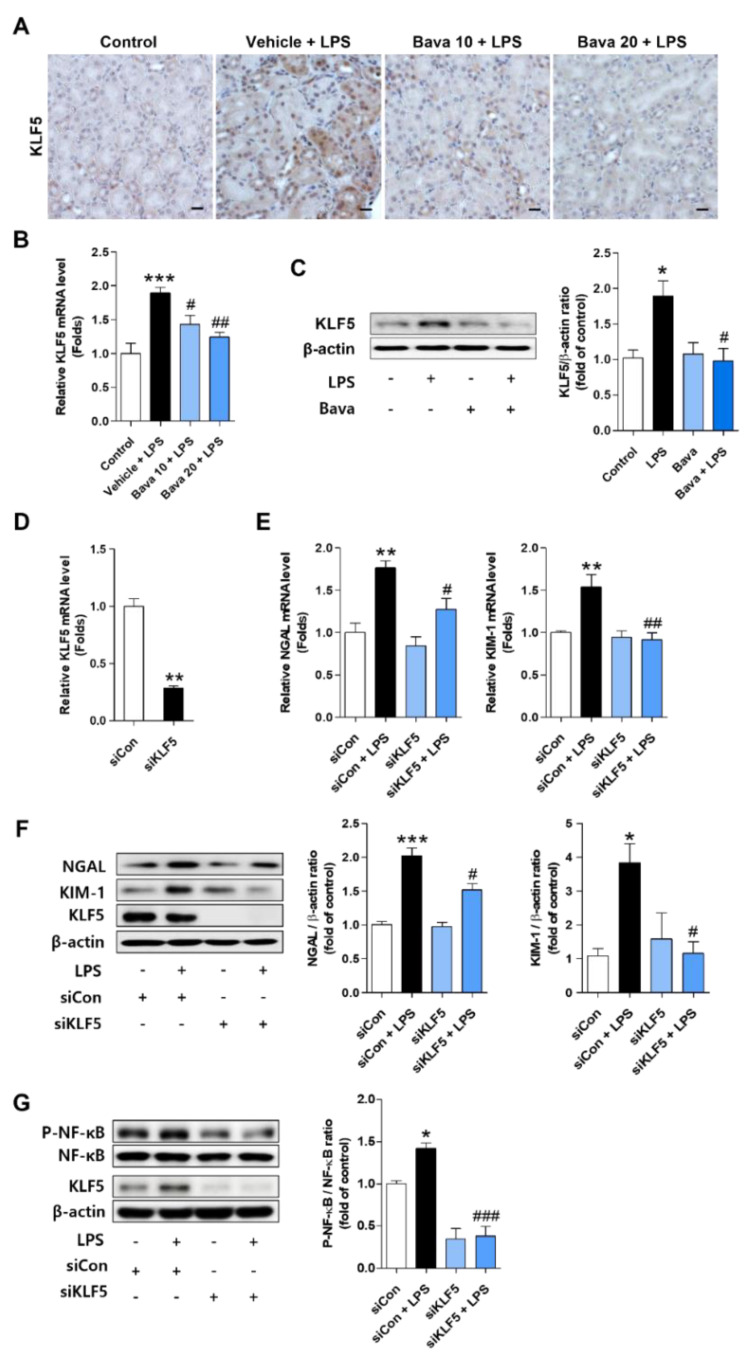
Bavachin decreases the expression level of kidney injury markers and NF-κB signaling via down-regulation of KLF5 in LPS-induced AKI mice and LPS-treated HK-2 cells. (**A**) Immunohistochemical detection of Krüppel-like factor 5 (KLF5) in kidney tissues using DAB. Nuclei were counterstained with hematoxylin (original magnification, 200×; scale bars, 20 μm; *n* = 3 mice per group). (**B**) The mRNA levels of KLF5 in kidney tissues were analyzed by qRT-PCR (*n* = 6 mice per group). (**C**) HK-2 cells were pretreated with 0.1 μg/mL bavachin for 1 h and then treated with LPS for 1.5 h. The protein level of KLF5 was analyzed by Western blotting, quantified using ImageJ software, and normalized to β-actin. (**D**–**G**) HK-2 cells were transfected with scrambled siRNA (siCon) and KLF5 siRNA (siKLF5) for 6 h and then treated with 1 μg/mL LPS for another 1.5 or 15 h. (**D**) The knockdown efficiencies of KLF5 siRNA in HK-2 cells were evaluated by qRT-PCR analysis. (**E**) The mRNA and (**F**) protein levels of NGAL and KIM-1 were analyzed using qRT-PCR and Western blotting, respectively. The bands were quantified using ImageJ software and normalized to β-actin (*n* = 3 or 4). (**G**) The protein levels of P-NF-κB were analyzed by Western blotting, quantified using ImageJ software, and normalized to NF-κB (*n* = 3). All data are presented as mean ± SEM. * *p* < 0.05, ** *p* < 0.01, *** *p* < 0.005 vs. control or siCon; ^#^ *p* < 0.05, ^##^ *p* <0.01, ^###^ *p* < 0.005 vs. vehicle + LPS or LPS or siCon + LPS.

The transcription factor KLF5 has been identified in the kidney and has been reported to regulate pathological conditions in various kidney cells [28]. As the role of KLF5 in the septic AKI model is unknown, we examined its involvement in the LPS-induced AKI model. As shown in Figure 6A,B, significantly higher levels of KLF5 mRNA and protein were detected in the kidneys of LPS-injected mice than in control mice (Figure 6A,B), which is consistent with the findings from LPS-treated HK-2 cells (Figure 6C). However, LPS-induced KLF5 expression was dose-dependently decreased in both mice and HK-2 cells by bavachin pretreatment (Figure 6A–C). Next, we transfected HK-2 cells with KLF5 siRNA and cultured them in the presence or absence of LPS to investigate the role of KLF5 in LPS-induced kidney injury marker expression and related signaling. We confirmed the knockdown of KLF5 expression in KLF5-transfected cells (Figure 6D). KLF5 knockdown attenuated LPS-stimulated NGAL and KIM-1 upregulation at both the mRNA and protein level (Figure 6E,F). Moreover, LPS-induced p-NF-κB was inhibited in KLF5 siRNA-transfected cells compared with that in scrambled siRNA-transfected cells (Figure 6G). These results suggest that the protective effect of bavachin against LPS-induced kidney injury is caused by reduced activation of NF-kB due to inhibition of KLF5 expression.

## 4. Discussion

Sepsis is defined as organ dysfunction caused by dysregulation of the host response in response to infection [1]. The risk of mortality in patients with sepsis-associated AKI is six to eight times higher than that in patients with sepsis [29]. Sepsis is the most common cause of AKI in critically ill patients, and complex pathophysiological mechanisms, such as apoptosis, oxidative stress, and inflammation, are involved [30]. While there are no specific treatments for septic AKI, early antibiotic administration and avoidance of hypotension and fluid overload can minimize risk [31]. AKI is characterized by a sharp decline in renal function and is diagnosed based on increased levels of blood creatinine and urea nitrogen as well as decreased daily urine output [4,6,32]. Moreover, the kidneys undergo changes, such as microvascular dysfunction and tubular damage [30].

Bavachin is a flavonoid component of the fruit of *Psoralea corylifolia* L. and has pharmacological benefits, including anti-inflammatory, antioxidant, and anti-apoptotic properties [14,33]. However, its effects on sepsis-associated AKI remain unknown; thus, we investigated whether bavachin can improve LPS-induced AKI and demonstrated the underlying mechanisms using in vivo and in vitro models. We found that bavachin administration significantly inhibited LPS-induced pathophysiological damage in the kidney.

NGAL is a 25 kDa protein of the lipocalin superfamily of carrier proteins [34] and is rapidly upregulated in tubular epithelial cells during kidney injury owing to various stimuli, such as ischemia/reperfusion [35]. KIM-1 is also expressed in tubular epithelial cells after ischemic or toxic injury [36,37]. Bavachin pretreatment reduced the expression of NGAL and KIM-1 in both LPS-induced AKI mice and LPS-treated HK-2 cells, suggesting that bavachin exerts a protective effect against LPS-induced acute tubular injury.

ROS are produced by endogenous sources or infiltrating cells in damaged kidneys and cause AKI or progressive renal damage [38]. Therefore, endogenous scavengers of ROS can attenuate renal tubular or vascular injury [39]. In this study, bavachin administration inhibited LPS-induced ROS production and NAC pretreatment attenuated NGAL and KIM-1 levels in LPS-treated cells, suggesting that oxidative stress is a potential target for the prevention of AKI by bavachin. NOX4 is expressed in proximal tubules and generates superoxide anions and hydrogen peroxide, known to be major ROS [40], suggesting that NOX4 plays an important role in the pathogenesis or development of LPS-induced AKI. It is well known that PKC, phosphoinositide 3-kinase, ERK, mothers against decapentaplegic homolog, and AMP-activated protein kinase pathways are important for the activation of NOX4 in various kidney diseases [25,41,42,43]. To investigate the mechanisms involved in the regulation of NOX4 expression by bavachin treatment, we determined the phosphorylation of these proteins and found that the LPS-induced increase in P-PKCβ and NOX4 expression was inhibited by bavachin pretreatment in vivo and in vitro. Xia et al. demonstrated that NOX4-dependent ROS generation in high glucose-treated mesangial cells is dependent on PKC activation [44,45]. LPS-induced apoptosis or methylglyoxal-induced inflammation is also mediated by PKC/ROS signaling pathways in renal tubular cells [45,46]. Yang et al. also demonstrated that LPS-induced oxidative stress in rat tubular cells is primarily attributed to PKC activation [47]. These results indicate that the inhibition of the P-PKCβ/NOX4 pathway might be involved in the protective effect of bavachin on LPS-induced ROS production in renal tubular cells.

Excessive production of proinflammatory cytokines in LPS-induced septic AKI is an important pathogenic factor. Clinical studies have shown that increased protein levels of proinflammatory cytokines, such as TNF-α, IL-1β, and IL-6, are observed in the blood of AKI patients [48]. Moreover, LPS can stimulate severe inflammatory reactions, resulting in the release of several inflammatory cytokines in HK-2 cells [49]. In this study, we found that LPS upregulated the levels of the inflammatory cytokines IL-1β, IL-6, and TNF-α in the serum and kidney tissues of mice and in HK-2 cells, and bavachin pretreatment attenuated this upregulation. NF-κB is highly activated at sites of inflammation in kidney disease [50] and can induce the transcription of proinflammatory cytokines and chemokines. In tubular epithelial-specific NF-κB knockout mice, a decrease in NF-κB activation protects mice from ischemic AKI by reducing apoptosis and chemokine expression [51]. We showed that LPS stimulation resulted in NF-κB activation and bavachin inhibited the LPS-mediated activation of NF-κB in HK-2 cells. These findings indicate that inactivation of NF-kB partly underlies the anti-inflammatory effect of bavachin in LPS-stimulated HK-2 cells.

The significance of MAPKs in the transcriptional regulation of LPS-induced production of inflammatory mediators has been previously demonstrated [52,53]. Moreover, stimulation of the renal tubules by LPS significantly increases the production of ROS and, thereby, the activation of MAPKs [54,55]. In our experiments, pretreatment with bavachin decreased the phosphorylation of MAPKs, especially of P38, ERK, and JNK. Lim et al. also demonstrated that LPS induces proinflammatory cytokine production through increased expression of the MAPK/NF-κB pathway [56]. Therefore, the suppressive effect of bavachin on MAPKs’ activation appears to contribute to the inhibition of inflammatory responses.

The transcription factor KLF5 is a member of the Krüppel-like factors (KLFs) family [57]. KLF5 was originally known as ‘basic transcription element-binding protein 2′, ‘colon KLF’, or ‘intestinal enriched KLF’ [58,59]. A recent study has reported that KLF5 controls podocyte apoptosis, kidney cell proliferation, tubulointerstitial inflammation, and kidney fibrosis in renal diseases [28]. In this study, the role of KLF5 in AKI was identified and the expression levels of KLF5 were increased in the kidney tissues of LPS-injected mice and LPS-treated HK-2 cells and suppressed by bavachin pretreatment. Moreover, KLF5 knockdown inhibited the activation of NF-κB and, consequently, reduced the expression of LPS-induced kidney injury markers, suggesting a correlation between KLF5 and NF-κB in the AKI model. Chen et al. reported that KLF5 induces the expression of proinflammatory cytokines such as TNF-α, IL-1β, and IL-6 by activating the NF-κB pathway [60]. In contrast, 17β-oestradiol inhibited the KLF5-NFκB inflammatory pathway in an Alzheimer’s disease mouse model [61]. Chanchevalap et al. demonstrated that KLF5 was activated by P38, ERK, and JNK and that KLF5 levels were reduced by treatment with each MAPK inhibitor in LPS-treated intestinal epithelial cells [62]. Although we did not investigate whether KLF5 is directly regulated by MAPKs in HK-2 cells in our study, the anti-inflammatory effect of bavachin was mediated by inhibition of KLF5-NF-κB signaling. Thus, the inhibition of MAPKs’ activation might contribute to the reduction in KLF5 expression, and these effects consequently result in the restored kidney function in the AKI model.

Our results suggest that this compound may exert beneficial effects in healthy patients to prevent kidney damage in people at risk of sepsis. Further experiments in the AKI model are required to prove the treatment effect of bavachin.

## 5. Conclusions

**Figure 7:**
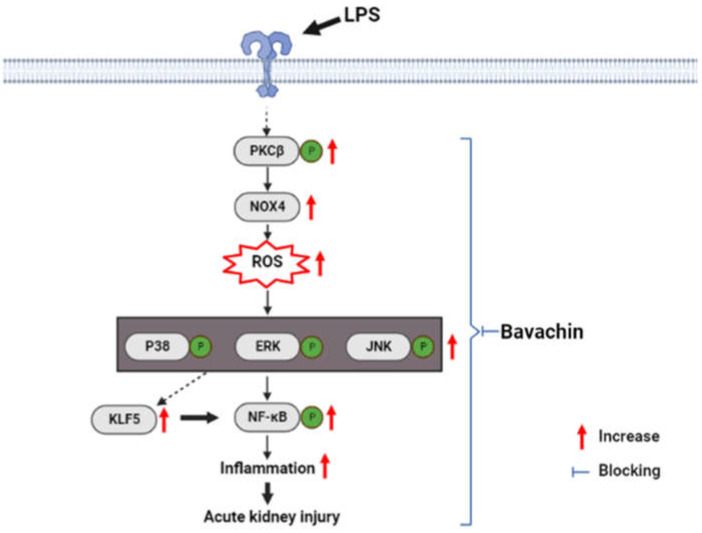
Schematic of the mechanism by which bavachin ameliorated acute kidney injury induced by LPS through PKCβ/MAPK/KLF5 signaling.

Our study revealed the protective effects of bavachin on septic AKI and the involvement of the PKCβ/MAPK/KLF5 signaling pathway (Figure 7). Bavachin treatment reduced LPS-induced oxidative stress and the inflammatory response during AKI progression; therefore, regulating these pathways using bavachin may be a potent strategy for the prevention and treatment of sepsis-induced AKI.

## Figures and Tables

**Table 1:** Primer sequence (human (h) and mouse (m)).

Gene	Accession Number	Primer Sequence
*hCyclophilin*	NM_000942	Forward: TGCCATCGCCAAGGAGTAG
		Reverse: TGCACAGACGGTCACTCAAA
*h18s RNA*	M10098	Forward: GCCGCTAGAGGTGAAATTCTTG
		Reverse: CATTCTTGGCAAATGCTTTCG
*hKLF5*	NM_001730	Forward: CTTCCACAACAGGCCACTTACTT
		Reverse: TCTGGAGCATCTCTGCTTGTC
*hHavcr1(KIM-1)*	NM_012206.3	Forward: CTTCACCTCAGCCAGCAGAAAC
		Reverse: GCCATCTGAAGACTCTGTCACG
*hLcn2(NGAL)*	NM_005564.5	Forward: TTTTGTTCCAGGTTGCCAGC
		Reverse: CTCACCACTCGGACGAGGTA
*hNOX4*	NM_001143836.3	Forward: TGTGCCGAACACTCTTGGC
		Reverse: ACATGCACGCCTGAGAAAATA
*hIL-1β*	NM_000576.2	Forward: GCTGCTCTGGGATTCTCTTCA
		Reverse: TGGCGAGCTCAGGTACTTCTG
*hIL-6*	NM_000600.4	Forward: GTACATCCTCGACGGCATCTC
		Reverse: GTGCCTCTTTGCTGCTTTCAC
*hTNF-α*	NM_000594.3	Forward: GAGATCAATCGGCCCGACTA
		Reverse: ACAGGGCAATGATCCCAAAG
*mCyclophilin*	NM_011149	Forward: TGGAGAGCACCAAGACAGACA
		Reverse: TGCCGGAGTCGACAATGAT
*mKLF5*	NM_009769	Forward: CACCCCACCTCCGTCCTAT
		Reverse: GGGTTGTGAATCGCCAGTTT
*mLcn2(NGAL)*	NM_008491.1	Forward: GGCAGCTTTACGATGTACAGCA
		Reverse: TCTGATCCAGTAGCGACAGCC
*mHavcr1(KIM-1)*	NM_134248.2	Forward: GCATCTCTAAGCGTGGTTGC
		Reverse: TCAGCTCGGGAATGCACAA
*mIl-1β*	NM_008361	Forward: CTACAGGCTCCGAGATGAACAAC
		Reverse: TCCATTGAGGTGGAGAGCTTTC
*mIl-6*	NM_031168.2	Forward: TCCATCCAGTTGCCTTCT
		Reverse: GGAGTGGTATCCTCTGTGAA
*mTNF-α*	NM_013693.3	Forward: CCAACGGCATGGATCTCAAAGACA
		Reverse: AGATAGCAAATCGGCTGACGGTGT

## Data Availability

The data used to support the findings of this study are available from the corresponding author upon request.

## Supplementary Materials

The following supporting information can be downloaded at: https://www.mdpi.com/article/10.3390/antiox11112096/s1, Figure S1: Bavachin decreases the expression of tubular injury markers by inhibiting ROS production in LPS-treated HK-2 cells.
