# Supplementary material for: Prevention of LPS-Induced Acute Kidney Injury in Mice by Bavachin and Its Potential Mechanisms
Source: Antioxidants (Basel). 2022 Oct 24;11(11):2096. doi: 10.3390/antiox11112096 (PMC9686515; doi:10.3390/antiox11112096)
Supplement: Supplementary file 1 [file antioxidants-11-02096-s001.zip › antioxidants-1950687-supplementary.pdf]

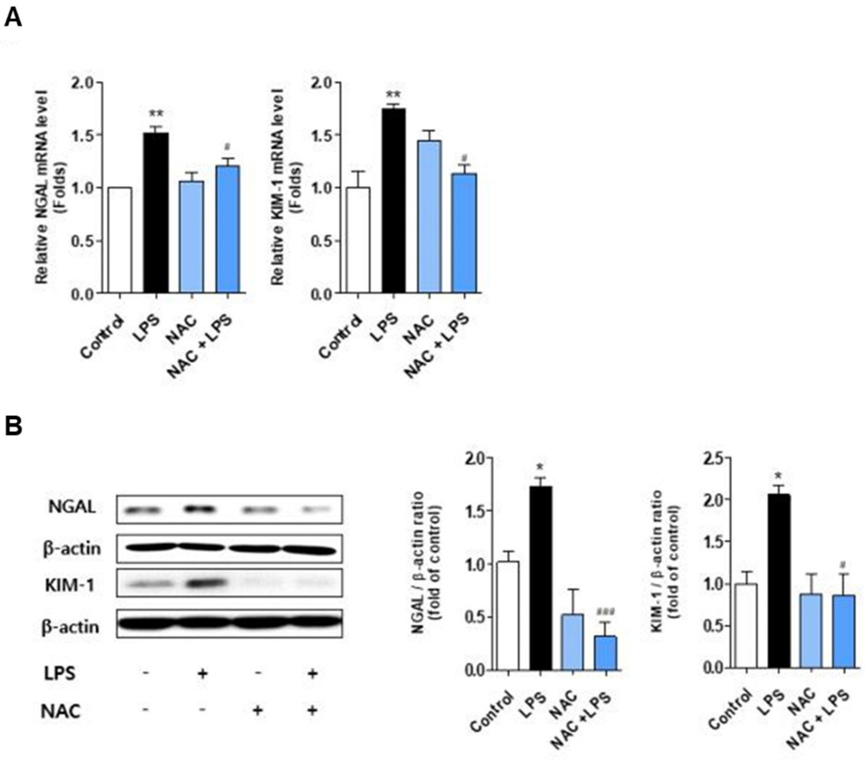

Supplementary Figure S1. Bavachin decreases the expression of tubular injury markers by inhibiting ROS production in LPS-treated HK-2 cells.

(**A-B**) HK-2 cells were pretreated with 1 mM N-acetyl-cysteine (NAC) for 1 h and then treated with 1 μg/mL LPS for 15 h. (**A**) mRNA levels of NGAL and KIM-1 were analyzed by qRT-PCR (n=3). (**B**) Protein levels of NGAL and KIM-1 were analyzed by western blotting, quantified using ImageJ software, and normalized to β-actin (n=3). Data are presented as mean ± SEM. *p<0.05, **p<0.01 vs. Control; $^{\#}$p<0.05, $^{\#\#\#}$p<0.005 vs. LPS.
